# Long-term performance of instrumental activities of daily living in young and middle-aged stroke survivors—Impact of cognitive dysfunction, emotional problems and fatigue

**DOI:** 10.1371/journal.pone.0216822

**Published:** 2019-05-16

**Authors:** Charlotte Blomgren, Hans Samuelsson, Christian Blomstrand, Christina Jern, Katarina Jood, Lisbeth Claesson

**Affiliations:** 1 Department of Health and Rehabilitation, Institute of Neuroscience and Physiology, the Sahlgrenska Academy at University of Gothenburg, Gothenburg, Sweden; 2 Region Västra Götaland, Södra Älvsborg Hospital, Dept of Occupational therapy, Borås, Sweden; 3 Department of Psychology, Faculty of Social Sciences at University of Gothenburg, Gothenburg, Sweden; 4 Department of Clinical Neuroscience, Institute of Neuroscience and Physiology, the Sahlgrenska Academy at University of Gothenburg, Gothenburg, Sweden; 5 Stroke Centre West, the Sahlgrenska Academy at University of Gothenburg, Gothenburg, Sweden; 6 Department of Laboratory Medicine, Institute of Biomedicine, the Sahlgrenska Academy at University of Gothenburg, Gothenburg, Sweden; 7 Department of Neurology, the Sahlgrenska University Hospital, Gothenburg, Gothenburg, Sweden; Universita degli Studi di Napoli Federico II, ITALY

## Abstract

**Background:**

With an upward trend in the number of people who return home to independent living after stroke, the ability to perform more complex activities is becoming an increasingly important long-term outcome after stroke. Although associations between Instrumental Activities of Daily Living (IADL) and cognitive dysfunction, emotional problems, and fatigue have been reported, less is known about the long-term impact of these stroke consequences on the performance of everyday activities in young and middle-aged stroke survivors.

**Objective:**

To explore the impact of cognitive dysfunction, emotional problems, and fatigue on long-term performance of instrumental activities of daily living in young and middle-aged stroke survivors

**Method:**

Data on stroke survivors, aged 18–69 at index stroke, were collected from the Sahlgrenska Academy Study on Ischaemic Stroke. IADL outcome was assessed using the Frenchay Activities Index (FAI), and the impact of chosen variables was assessed using Spearman´s rank-order correlation and logistic regression.

**Results:**

Seven years after index stroke, 296 stroke survivors (median age of 64) were included in this study. Cognitive dysfunction showed the strongest correlations with FAI outcome and independently explained worse outcome on FAI summary score and the domain of work/leisure activities. Fatigue was independently explanatory of worse outcome on FAI summary score and domestic chores, while depressive symptoms independently explained worse outcome on work/leisure activities. In a subgroup with only those participants who had no or minimal residual neurological deficits at follow-up (NIHSS score 0), cognitive dysfunction independently explained worse outcome on FAI summary score and work/leisure activities. Depressive symptoms independently explained worse outcome on FAI summary score and domestic chores.

**Conclusion:**

Our results show that in young and middle-aged stroke survivors, cognitive dysfunction, depressive symptoms, and fatigue negatively impact performance of IADL even at seven years post stroke onset. Further, we have shown that an impact of both cognitive dysfunction and depressive symptoms can be found also among stroke survivors with mild or no remaining neurological deficits.

## Introduction

Currently, there is an upward trend in the number of people who return home to independent living after stroke [1]. For those, it is important not only to resume basic activities of daily living, but also to be able to perform more complex activities in everyday life. Thus, the ability to perform more complex activities is becoming an increasingly important long-term outcome after stroke. In this study we use the term Instrumental Activities of Daily Living (IADL) to refer to the more complex activities, including domestic chores, social activities, and gainful work [2]. IADLs are activities that typically must be performed for the stroke survivor to continue living in the community [3]. Long-term follow-up studies indicate that stroke can have a negative impact on the ability to perform IADL also many years later [1, 4–6]. However, long-term studies including IADL outcomes are still scarce and few focus on young and middle-aged stroke survivors. Around one third of ischaemic strokes occur in those under the age of 70 years [7]. For these younger stroke survivors, the responsibilities of family life and work, as well as the demands from society differ from those of older stroke survivors [8–11].

The Sahlgrenska Academy Study on Ischaemic Stroke (SAHLSIS) has previously reported reduced levels of participation in IADL in young and middle-aged stroke survivors seven years post stroke onset [12]. In our sample of consecutive patients, the majority of participants had mild or no residual neurological impairments at follow-up. Although showing less outwards signs of stroke related disability, this group is reported to experience deficits when returning to the lives they were living before stroke [13]. IADLs are activities that generally require higher cognitive and social skills [14, 15]; consequently, associations with cognitive dysfunction [14, 16, 17], emotional problems [14, 18], and fatigue [19–22] have been reported. Currently, less is known about the long-term consequences of these associations for everyday activities in young and middle-aged stroke survivors. A high prevalence of cognitive dysfunction (89%) at long-term follow-up has previously been reported for participants in SAHLSIS [23] but associations with IADL have not yet been studied. Thus, we aimed to explore the impact of cognitive dysfunction, emotional problems, and fatigue on long-term performance of instrumental activities of daily living in young and middle-aged stroke survivors.

## Material and methods

### Participants

Data on stroke survivors were collected as part of the Sahlgrenska Academy Study on Ischaemic stroke (SAHLSIS), the design of which has been described in more detail elsewhere [24]. The study comprises adult patients presenting with a first-ever or recurrent acute ischaemic stroke before the age of 70 years. Participants were consecutively recruited when seeking medical care at the stroke unit of Sahlgrenska University hospital in Gothenburg, Sweden from 1998 to 2003 and then prospectively followed. SAHLSIS included participants with all levels of stroke severity. Recurrent strokes and deaths during follow-up have been identified using the Swedish Cause of Death Register, the National Patient register, and medical records as previously described [25]. As previously described in detail [12], all surviving participants (n = 358) were invited to participate in a comprehensive assessment seven years after index stroke. The follow-up study protocol included a postal questionnaire and two study visits; 1) to a study nurse trained in the use of the instruments and 2) to a study neurologist. Patients who were unable to visit the clinic were offered a home visit.

### Ethics

Written informed consent was obtained from all participants. For participants who were unable to communicate, consent was obtained from their next-of-kin. The study was approved by the Regional Ethical Review Board in Gothenburg.

### Instruments

The Frenchay Activities Index (FAI) [26] was used to assess outcome in IADL. The 15 items of the FAI cover a range of complex activities, including domestic, work, and social activities that require some decision-making and organisation on behalf of the patient. The FAI consists of a single summary score, ranging from a 0 (inactive) to 45 (highly active), which can be divided into subscale scores. The item scoring is based upon the frequency with which an activity is performed. Based on research by Lin et al. [27] that suggests that the FAI be divided into two subscales, in order to minimise the risk of items overlapping, we have in a previous study [12] made the decision to use two rather than three subscales for the FAI. The two domains recommended by Lin et al. [27] and used along with the summary score for analysis also in this study are domestic chores and work/leisure. The questions were asked by the research nurse and participants rated their frequency of performance on each item during the interview.

Neurological deficit was assessed using the National Institute of Health Stroke Scale (NIHSS) [28]. Scores range from 0–42, with 0 indicating better outcome. In this study, a cut-off level was set at 0. This cut-off was used to capture those with no or minimal remaining neurological deficits at the follow-up neurological examination.

The Fatigue Impact Scale (FIS)[29] was used to assess fatigue in relation to daily activities. The FIS consist of 40 items, intended to reflect activities and common situations in everyday life where fatigue can have a profound impact. Scores range from 0–160 with higher scores indicating a higher degree of impact of fatigue.

Cognitive functions were screened using a Swedish translation of the Barrow Neurological Institute Screen for higher cerebral functions (BNIS) that has previously been validated [30, 31] and has shown good screening qualities for cognitive dysfunction in this particular population [23]. The total score (maximum 50 points) reflects the overall cognitive function.

Symptoms of anxiety and/or depression were assessed using a Swedish version of the Hospital Anxiety and Depression Scale (HAD) [32]. The HAD consists of one subscale for anxiety and one for depression, which are summarised separately. Each scale ranges from 0–21, with higher scores indicating inferior mood.

All the above assessments relevant to analyses were carried out at the seven-year follow-up.

### Data analysis

We have previously reported that gender, cohabitation status, and initial stroke severity are predictive of IADL outcome in this population, and that age showed a univariable correlation with FAI outcome [12]. For this study, with a cross-sectional design, we included age at follow-up, gender, cohabitation status, and follow-up score on the NIHSS as background variables in analyses. For tests between groups, Mann-Whitney U test was used for continuous variables and Fisher´s exact test for dichotomous variables. Univariable correlations between FAI, explanatory variables, and background variables were studied using Spearman´s rank-order correlation (r_s_). A *r*_*s*_ ± .10–.29 was considered a weak correlation, *r*_*s*_ ± .30–.49 a moderate correlation, and *r*_*s*_ ≥ ± .50 was considered a strong correlation[33]. Since the FAI is not normally distributed in this sample, the FAI was dichotomised in order to perform multiple regression analyses. In order to more easily compare the univariable and multivariable results, a univariable logistic regression was performed, in addition to Spearman´s rank-order correlation. Worse outcome was defined as a score below the study population median. Scores of explanatory variables were treated as continuous variables. Score on the FIS, ranging from 0–160, was rescaled by dividing the score with 5, thereby allowing for a more adequate comparison of OR with other outcome instruments. Explanatory and background variables with a significant Spearman´s rank order correlation with FAI summary or domain scores were included in a multivariable stepwise forward logistic regression in order to select variables that were independently explanatory of worse outcome on the FAI. Based on strong univariable correlations between FAI outcome and NIHSS score, a sub-analysis was carried out using only results of participants with a NIHSS score of 0. This was done in order to explore if explanatory variables had a similar explanatory value regarding FAI outcome also for those participants with no or minimal remaining neurological deficits at the follow-up neurological examination. Odds ratios (OR) with 95% confidence intervals and p-values were obtained, and the area under the ROC curve was given for the model for a description of the model´s goodness of fit. All significance tests were two-sided and conducted at the 5% significance level. Data were analysed using SPSS version 22.0 [34].

## Results

**Fig 1:**
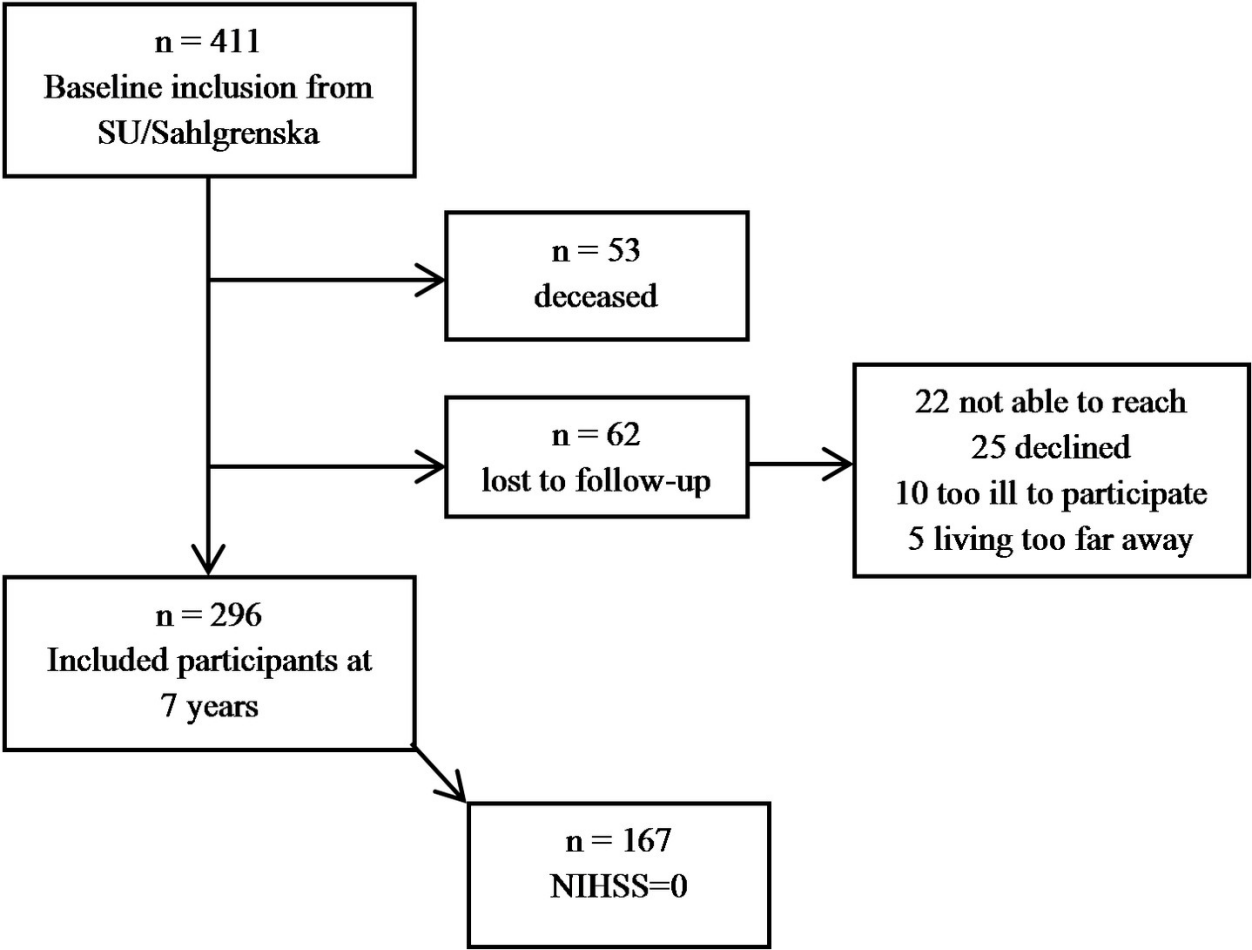
Flowchart of patient inclusion.

Of the 411 participants included at baseline, 53 participants were deceased and a further 62 did not participate in the seven-year follow-up assessments, for reasons given in Fig 1. This leaves the total number of participants at 296. Mean time from index stroke to follow-up was 7.3 years (SD 0.25). The number of participants with at least one recurrent stroke during the follow-up period was 59 (20%). Mean systolic and diastolic blood pressure were 133(SD 20) mm Hg and 74 (SD 11) mm Hg, respectively. Coronary heart disease had been diagnosed in 8.6% and atrial fibrillation in 13.4% of the participants. Background data and scores on outcome and explanatory measures are shown in Table 1.

**Table 1:** Description of background, explanatory, and outcome variables at 7 years follow-up.

Background variables	Total sample	NIHSS = 0
	n = 296	n = 167
Age^▲^	61.9 (10.9)	60.8 (11.1)
	64.1 (56,9–69.5)	63.6 (55.7–68.9)
Gender -Male	184 (62.2%)	105 (62.9%)
Cohabitation status -Living alone	104 (35.1%)	48 (28.7%)
Outcome variables		
FAI summary score(min–max: 0–45)	24.1 (10.3)	28.72 (6.6)
	27 (18.3–32)	29 (26–33)
FAI domain of work/leisure(min-max: 0–27)	12.7 (6.0)	15.4 (4.4)
	13 (8–17)	16 (12–18)
FAI domain of domestic chores(min-max: 0–18)	11.37 (5.7)	13.3 (4.4)
	12.5 (8–16.8)	15 (11–17)
Explanatory variables		
BNIS(min-max: 0–50)^▲^	38.9 (6.6)	41.3 (4.9)
	39 (35–43)	41 (38–46)
FIS(min-max: 0–160)^▲^	39.3 (37.5)	30.4 (33.9)
	30 (5–66.7)	17 (2–48)
HAD-A(min-max: 0–21)^▲^	4.4 (4.2)	4.2 (4.1)
	3 (1–7)	3 (1–6)
HAD-D(min-max: 0–21)^▲^	4.5 (4.1)	3.6 (3.8)
	3 (1–7)	2.5 (1–5)
NIHSS(min-max: 0–42)^▲^	2.1 (4.1)	
	0 (0–2)	

Values given as mean (SD) and median (Q1-Q3) for continuous data and n (%) for nominal data

FAI, Frenchay Activities Index; BNIS, Barrow Neurological Institute Screen for higher cerebral functions

FIS, Fatigue Impact Scale; HAD, Hospital Anxiety (A) Depression (D) Scale

NIHSS, National Institute of Health Stroke Scale

^▲^Missing values total sample: Age n = 2; BNIS n = 11; HADA n = 8; HADD n = 8; FIS n = 41; NIHSS n = 5. Missing values NIHSS = 0: Age n = 1; BNIS n = 1; HAD n = 1; FIS n = 8

Frequency of performance for each of the FAI items is shown in S1 Table. The number of participants with missing data was higher for FIS than for the other instruments, n = 41 (14%). Reasons for missing data were as follows; 14 (4.7%) had declined, 1 (0.3%) was ill, 17 (5.7%) had not returned the questionnaire, and 9 (3%) had completed parts of the questionnaire but not the FIS. Participants with missing data on FIS had worse outcome on the FAI (p<0.001). At follow-up, 167 participants (56.4%) had a NIHSS score of 0 and were further analysed as a subgroup. Participants with a NIHSS score of 0 had significantly better scores (p <0.001) on all outcome measures except for the anxiety part of the HAD (p = 0.528) compared to participants with a NIHSS score of 1 or higher. There were no significant gender differences, but participants with a NIHSS score of 0 were younger (p <0.05) and more often living with another adult (p <0.01).

Results of the correlation analyses for the total sample are shown in Table 2 and results for the subsample with NIHSS score of 0 are shown in Table 3.

**Table 2:** Odds ratios and correlations between FAI, explanatory, and background variables for total sample, n = 296.

	FAI summary score					FAI work/leisure					FAI domestic chores				
	worse outcome = FAI score <27					worse outcome = FAI score <13					worse outcome = FAI score <12.5				
	Univariable correlation			Multivariable correlation		Univariable correlation			Multivariable correlation		Univariable correlation			Multivariable correlation	
				AUC 0.778					AUC 0.834					AUC 0.800	
Explanatory variables	r_s_	OR (95% CI)	AUC	OR (95% CI)	p-value	r_s_	OR (95% CI)	AUC	OR (95% CI)	p-value	r_s_	OR (95% CI)	AUC	OR (95% CI)	p-value
BNIS	0.56	0.83 (0.79–0.88)	0.753	0.89 (0.84–0.95)	<0.001	0.56	0.83(0.79–0.88)	0.756	0.89 (0.83–0.94)	<0.001	0.37	0.89 (0.85–0.93)	0.687		
	p <0.001					p <0.001					p <0.001				
FIS (/5)	-0.35	1.10 (1.06–1.15)	0.694	1.07 (1.03–1.11)	0.002	-0.38	1.10 (1.06–1.14)	0.684			-0.20	1.04 (1.01–1.07)	0.573	1.06 (1.01–1.10)	0.014
	p <0.001					p <0.001					p 0.002				
HAD-A	-0.06	1.04 (0.98–1.10)	0.538			-0.12	1.07 (1.01–1.13)	0.553			0.01	0.97 (0.92–1.03)	0.532		
	p 0.29					p 0.042					p 0.88				
HAD-D	-0.34	1.18 (1.10–1.26)	0.681			-0.36	1.18 (1.10–1.26)	0.671	1.10 (1.02–1.19)	0.016	-0.24	1.07 (1.01–1.13)	0.590		
	p <0.001					p <0.001					p <0.001				
Background variables															
Age	-0.26	1.02 (1.00–1.05)	0.581			-0.24	1.03 (1.00–1.05)	0.599			-0.23	1.04 (1.01–1.06)	0.614	1.03 (1.0–1.06)	0.035
	p <0.001					p <0.001					p <0.001				
Gender male	-0.06	1.15 (0.72–1.85)	0.517			0.18	0.54 (0.33–0.87)	0.573	0.46 (0.24–0.86)	0.015	-0.31	3.74 (2.26–6.17)	0.649	6.76 (3.35–13.67)	<0.001
	p 0.35					p 0.002					p <0.001				
Living alone	-0.06	1.08 (0.67–1.75)	0.510			-0.25	3.04 (1.85–4.99)	0.625	2.28 (1.22–4.26)	0.010	0.12	0.58 (0.36–0.95)	0.561	0.33 (0.17–0.66)	0.002
	p 0.30					p <0.001					p 0.035				
NIHSS	-0.58	1.64 (1.37–1.96)	0.758	1.29 (1.08–1.54)	0.005	-0.59	1.64 (1.38–1.96)	0.769	1.39 (1.17–1.65)	<0.001	-0.43	1.26 (1.14-1-38)	0.670	1.22 (1.09–1.38)	0.001
	p <0.001					p <0.001					p <0.001				

FAI, Frenchay Activities Index; BNIS, Barrow Neurological Institute Screen for higher cerebral functions

FIS, Fatigue Impact Scale; NIHSS, National Institute of Health Stroke Scale

HAD, Hospital Anxiety (A) Depression (D) Scale

AUC, area under curve

Spearman´s rank-order correlation was carried out treating FAI as a continuous variable.

For information regarding missing values, see Table 1

**Table 3:** Odds ratios and correlations between FAI, explanatory, and background variables for participants with NIHSS score of 0, n = 167.

	FAI summary score					FAI work/leisure					FAI domestic chores				
	worse outcome = FAI score <29					worse outcome = FAI score <16					worse outcome = FAI score <15				
	Univariable correlation			Multivariable correlation		Univariable correlation			Multivariable correlation		Univariable correlation			Multivariable correlation	
				AUC 0.728					AUC 0.718					AUC 0.812	
Explanatory variables	r_s_	OR (95% CI)	AUC	OR (95%CI)	p-value	r_s_	OR (95% CI)	AUC	OR (95%CI)	p-value	r_s_	OR (95% CI)	AUC	OR (95%CI)	p-value
BNIS	0.31	0.85 (0.79–0.91)	0.702	0.85 (0.79–0.91)	<0.001	0.29	0.88 (0.82–0.94)	0.669	0.85 (0.790.92)	<0.001	0.10	0.98 (0.92–1.05)	0.539		
	p <0.001					p <0.001					p 0.20				
FIS (/5)	-0.13	1.03 (0.99–1.08)	0.537			-0.13	1.04 (1.00–1.09)	0.567			-0.06	1.03 (0.99–1.08)	0.545		
	p 0.10					p 0.09					p 0.44				
HAD-A	-0.07	1.05 (0.97–1.13)	0.538			-0.06	1.05 (0.97–1.13)	0.535			-0.04	1.01 (0.94–1.09)	0.543		
	p 0.35					p 0.45					p 0.66				
HAD-D	-0.23	1.11 (1.02–1.21)	0.583	1.11 (1.01–1.22)	0.035	-0.18	1.09 (1.00–1.18)	0.578			-0.17	1.07 (0.98–1.16)	0.596	1.16 (1.04–1.29)	0.006
	p 0.003					p 0.024					p 0.027				
Background variables															
Age	-0.20	1.02 (0.99–1.05)	0.586			-0.19	1.04 (1.01–1.08)	0.648			-0.09	1.02 (0.99–1.05)	0.538		
	p 0.012					p 0.012					p 0.27				
Gender male	-0.11	1.73 (0.91–3.28)	0.563			0.24	0.42 (0.22–0.79)	0.601	0.29 (0.14–0.59)	0.001	-0.454	5.07 (2.53–10.15)	0.677	5.86 (2.60–13.17)	<0.001
	p 0.15					p 0.002					p <0.001				
Living alone	0.11	0.71 (0.36–1.41)	0.534			-0.20	2.00 (1.01–3.95)	0.571			0.35	0.14 (0.06–0.32)	0.678	0.12 (0.05–0.30)	<0.001
	p 0.17					p 0.011					p 0.011				

FAI, Frenchay Activities Index; BNIS, Barrow Neurological Institute Screen for higher cerebral functions

FIS, Fatigue Impact Scale; HAD, Hospital Anxiety (A) Depression (D) Scale

NIHSS, National Institute of Health Stroke Scale

AUC, area under curve

Spearman´s rank-order correlation was carried out treating FAI as a continuous variable.

For information regarding missing values, see Table 1

### Total sample

In univariable analyses, BNIS score showed strong univariable correlation with FAI summary score and work/leisure activities and a moderate correlation with domestic chores. HAD-A score showed weak correlation with work and leisure activities and no significant correlation with FAI summary score or domestic chores. HAD-D score showed a moderate correlation with FAI summary score and work/leisure activities, and a weak correlation with domestic chores. Score on the FIS was moderately correlated with FAI summary score and work/leisure activities and showed a weak, but significant correlation with domestic chores. In multivariable logistic regression analyses, BNIS score was independently explanatory of FAI summary score and work/leisure activities, such that increasing cognitive dysfunction increased the odds of a worse outcome. A higher score on the FIS was independently explanatory of a worse outcome on FAI summary score and domestic chores. A higher score on the HAD-D was explanatory of worse outcome within work/leisure activities. The area under the ROC curve for the multivariable models was 0.78 for FAI summary score, 0.83 for work/leisure activities, and 0.80 for domestic chores.

### Subgroup NIHSS score of 0

In the subgroup with only those participants who had no or minimal residual neurological deficits at follow-up (NIHSS score 0), BNIS score was moderately correlated with FAI summary score and showed weak correlation with work/leisure activities. No significant correlation was found between BNIS score and domestic chores. The HAD-D score showed weak but significant correlation with both summary score and the two domain scores of FAI. Scores on FIS and on HAD-A did not show significant correlation with any of the FAI outcomes and were not included in multivariable analyses. In multivariable logistic regression analyses, BNIS score was independently explanatory of FAI summary score and work/leisure activities. HAD-D score was independently explanatory of worse outcome on FAI summary score and domestic chores. The area under the ROC curve for the multivariable models was 0.73 for FAI summary score, 0.72 for work/leisure activities, and 0.81 for domestic chores.

## Discussion

The results from this study show that cognitive dysfunction, emotional problems, and fatigue have an impact on long-term performance of IADL in this population of young and middle-aged stroke survivors. The impact was generally higher for the more complex activities and for participants who also showed residual neurological deficits.

With a high prevalence of cognitive dysfunction previously reported in this sample [23], this study adds to these results by showing a significant impact on IADL outcome. Score on the BNIS was the variable having the strongest correlations with FAI outcome and significantly contributed to the model’s explanatory value with regard to outcome on FAI summary score and work/leisure activities. However, in multivariable analysis, including also demographic data and neurological outcome as background variables, BNIS was not independently explanatory of outcome on the FAI domain of domestic chores. Liman et al. [16] have previously reported a stronger association between FAI and a MMSE score ≤24_ad j_ than between MMSE and Barthel Index. Activities included in the Barthel Index are less complex than those in the FAI, and a greater impact within work/leisure activities than domestic chores may be due to the same difference in complexity. In a previous study on the influence of cognitive performance on long-term functional outcome after ischaemic stroke in young adults, Synhaeve et al. [35] found no clear relationship between long-term cognitive deficits and long-term IADL. Synhaeve et al. [35] used the Instrumental Activities of Daily Living scale to assess performance of more complex activities. In our study, the work/leisure domain of FAI include several more complex activities, such as gainful work and social outings, that are not included in the Instrumental Activities of Daily Living scale. These differences between measures of IADL can be an explanation for the stronger correlations between cognitive dysfunction and IADL outcome found in our study. The work/leisure domain of FAI included gainful work, and we have previously reported that just over 50% of participants, aged 65 or younger, never perform this activity [12]. A strong predictive value of cognitive severity on the ability to return to work has previously been found in young stroke patients with first-ever ischaemic stroke [36]. Although the FAI does not include many activities common in today`s increasingly complex society, results from our study, along with those of Kauranen et al. [36], show that cognitive dysfunction has an effect on the more complex IADLs.

Score on the Fatigue Impact Scale was significantly correlated with both of the FAI domains, indicating an impact of fatigue on IADL. The univariable correlations were stronger for work/leisure activities than domestic chores. However, in multivariable analyses, including also HAD depression score, BNIS and background variables, FIS was not independently explanatory of outcome in work/leisure activities. With previous studies reporting that survivors experience fatigue as the worst, or one of the worse sequelae of stroke [37–39], we had expected to find a greater impact of fatigue than indicated by the moderate to weak correlations found in this sample. One explanation is that fatigue may have a greater impact on activities not captured by the use of the FAI. Studying self-reported fatigue and associated factors six years after stroke, Elf. et al. [40] found no association between fatigue and the activities included in the FAI, although participants in their study perceived that fatigue did hinder daily activities. Crosby et al. found that as many as 64% of their sample reported fatigue as a frequent problem but still found no significant correlation between fatigue and instrumental activities. One plausible explanation is the use of IADL instruments, which typically only measure the frequency of performance in predefined activities. However, after seven years, it is also possible that participants have new priorities and learned to adapt their repertoire of activities according to their abilities, making the impact of fatigue less prominent and affecting the rating of fatigue on the FIS. Fatigue is a complex phenomenon and as such often difficult to define and separate from other symptoms. There are a number of potential factors that can influence fatigue [9, 41], depression perhaps being the most common. However, there is a growing body of research showing that post stroke fatigue can occur also in the absence of depression [39, 41–43], indicating that fatigue and depression should be explored as separate consequences of stroke. In our study, depressive symptoms showed univariable correlation with both FAI domains and was independently explanatory of outcome in work/leisure activities.

A confounding effect of depression for the association between fatigue and IADL in chronic stroke has previously been reported by van de Port [21]. Although fatigue and depressive symptoms correlated with IADL, none of the final models in our study found both fatigue and depressive symptoms to be independently explanatory when both were included in the same analysis. We did not find high enough correlations between BNIS, HAD and FIS to indicate problems with multicollinearity in the models. However, there is arguably a certain amount of overlap between these variables in our study. Cognitive dysfunction may affect ratings of both depression and fatigue. As we found cognitive dysfunction to be the strongest explanatory variable, this may contribute to the fact that fatigue did not have the effect on IADL that has been reported in other studies. Not surprisingly, this overlap varied depending on the type of activity carried out. There is likely a reciprocal relation between explored factors and IADL. By performing daily activities, stroke survivors can gradually discover their strengths and weaknesses [44]. Exploring consequences of 'hidden dysfunctions' one year after a mild stroke in person < 75 years, Carlsson et al. [45] found that as participants became confronted with their difficulties, many developed new priorities and gave up activities. A person no longer able to perform valued activities due to cognitive dysfunction may develop signs of depression, and a person who learns to adjust to fatigue may no longer experience fatigue as a problem in IADL.

Associations between cardiovascular factors and depression [46] and cognitive impairment [47, 48] have been reported. In our relatively younger cohort of stroke survivors however, neither systolic blood pressure, coronary heart disease, nor atrial fibrillation showed significant univariate correlation to FAI outcome. Liguori et al. [46] have described important reciprocal relationships between depression and chronic heart failure among elderly, However we did not find univariate correlations between coronary heart disease and the FAI, nor between coronary heart disease and depressive symptoms in our younger sample of stroke survivors.

Exploring if outcome variables had similar explanatory value regarding FAI outcome also for those participants with no or minimal remaining neurological deficits at follow-up, we found that, although lower, there were still significant correlations between BNIS, HAD depression score, and IADL outcome. Just as for the total sample, BNIS showed the strongest correlations and was independently explanatory of FAI summary score and of work/leisure activities. In a recent study exploring the long-term functional outcomes of stroke patients with very mild severity at 6 months after stroke, Chang et.al. [49] reported that 22.6% of patients with a NIHSS score of 0 showed mild to moderate cognitive impairment. Our results add to these results by showing also an impact on IADL. The findings of significant impact on long-term IADL of both cognitive dysfunction and depression show that mild stroke, according to the allocation provided by the NIHSS, does not mean that a person will not experience stroke-related problems upon returning to their everyday life activities.

### Limitations

For this study, a limitation of the project design is that longitudinal data concerning many of the outcome variables were not collected. The number of participants with missing data was higher for FIS than for the other instruments, i.e. n = 41 (14%). This instrument was distributed as a postal questionnaire, which may well have contributed to the higher number of missing data [50]. Participants with missing data on FIS had worse outcome on the FAI. Our findings therefore most likely represent an underestimation of the experienced long-term impact of fatigue.

This study also has several strengths one being its large sample of consecutively recruited stroke survivors. The response rate at the long-term follow-up was 83% (including all non-recurrent and recurrent strokes but not deaths). Information concerning loss to follow-up was recorded and available from baseline until the seven year follow-up. As previously discussed in more detail [12] the younger age of participants allows for long-term follow-up and the exploration of the effect of stroke on those previously living active lives. It also facilitates more reliable analyses due to less comorbidity. Based on the longer life expectancy in this group and different demands on younger and middle-aged stroke survivors compared to those of higher age, we believe this group is important to highlight when studying long-term effects.

## Conclusion

Our results show that in young and middle-aged stroke survivors, cognitive dysfunction, depressive symptoms and fatigue negatively impact performance of IADL as late as seven years post stroke onset. Further, we have shown that an impact of both cognitive dysfunction and depressive symptoms can be found also among stroke survivors with mild or no remaining neurological deficits. Cognitive impairment, post-stroke depression, and fatigue have been reported as being both potentially underdiagnosed and under-treated [9, 51]. Currently, there is limited evidence for the effectiveness of interventions targeting cognitive deficits, post stroke fatigue, and depression [52–54]. Focusing on their impact in everyday life may provide guidance in designing interventions that promote higher levels of IADL performance after stroke.

## Supporting information

S1 Table Long-term outcome on the Frenchay Activities Index. n = 296.
(PDF)
