# Supplementary material for: Long-term performance of instrumental activities of daily living in young and middle-aged stroke survivors—Impact of cognitive dysfunction, emotional problems and fatigue
Source: PLoS One. 2019 May 16;14(5):e0216822. doi: 10.1371/journal.pone.0216822 (PMC6522019; doi:10.1371/journal.pone.0216822)
Supplement: S1 Table — n = 296. (PDF) [file pone.0216822.s001.pdf]

Supporting information 1. *Long-term outcome on the Frenchay Activities Index. n =296*

Domain of domestic chores	mean (SD): 11.4 (5.7)<br>median (Q1-Q3): 12.5 (8-16.75)<br>min-max: 0-18			
	Never	Under once weekly	1-2 times a week	Most days
Preparing meals	93 (31.4%)	7 (2.4%)	70 (23.6%)	126 (42.6%)
Washing dishes	53 (17.9%)	6 (2%)	63 (21.3%)	174 (58.8%)
	Never	1-2 times in 3 months	3-12 times In 3 months	At least weekly
Washing clothes	125 (42.2%)	9 (3%)	43 (14.5%)	119 (40.2%)
Light housework	111 (37.5%)	7 (2.4%)	51 (17.2%)	127 (42.9%)
Heavy housework	98 (33.1%)	9 (3%)	34 (11.5%)	155 (52.4%)
Local shopping	56 (18.9%)	3 (1%)	11 (3.7%)	226 (76.4%)
Domain of Work/Leisure	mean (SD): 12.7 (6.0)<br>median (Q1-Q3): 13 (8-17)<br>min-max: 0-26			
	Never	1-2 times in 3 months	3-12 times in 3 months	At least weekly
Social outings	78 (26.4%)	52 (17.6%)	79 (26.7%)	87 (29.4%)
Walks	63 (21.3%)	5 (1.7%)	9 (3%)	219 (74 %)
Hobby/Sport	128 (43.2%)	7 (2.4%)	13 (4.4%)	148 (50%)
Driving a car/ Travel on bus	55 (18.6%)	19 (6.4%)	31 (10.5%)	191 (64.5%)
	Never	1-2 times in 6 months	3-12 times in 6 months	At least weekly
Outings/ Car rides	62 (20.9%)	89 (30.1%)	107 (36.1%)	38 (12.8%)
	None	Light	Moderate	All necessary
Gardening	177 (59.8%)	35 (11.8%)	34 (11.5%)	50 (16.9%)
Household and/or Car maintenance	186 (62.8%)	35 (11.8%)	49 (16.6%)	26 (8.8%)
	None	1 in 6 months	Less than 1 a fortnight	Over 1 a fortnight
Reading books	120 (40.5%)	52 (17.6%)	56 (18.9%)	68 (23%)
	None	Up to 10 h/week	10-30 h/week	Over 30 h/week
Gainful work	205 (69.3%)	6 (2%)	34 (11.5%)	51 (17.2%)
Summary score	mean (SD): 24.1 (10.3)<br>median (Q1-Q3): 27 (18.25-32)<br>min-max: 0-44			

Each item rated on a 0-3 scale representing lowest to highest actual participation in each activity
Proportions shown as n (%)
